# Optical and material analysis of opacified hydrophilic intraocular lenses after explantation: a laboratory study

**DOI:** 10.1186/s12886-015-0149-1

**Published:** 2015-11-25

**Authors:** Tamer Tandogan, Ramin Khoramnia, Chul Young Choi, Alexander Scheuerle, Martin Wenzel, Philipp Hugger, Gerd U. Auffarth

**Affiliations:** David J Apple International Laboratory for Ocular Pathology and International Vision Correction Research Centre (IVCRC), Department of Ophthalmology, University of Heidelberg, Im Neuenheimer Feld 400, 69120 Heidelberg, Germany; Eye Clinic Petrisberg, Trier, Germany, Max-Planck-Straße 16, 54296 Trier, Germany; Eye Clinic Esslingen, Esslingen, Germany, Augen-Praxis-Klinik-Esslingen Adlerstraße 6, 73728 Esslingen, Germany; Department of Ophthalmology, Kangbuk Samsung Hospital, Sungkyunkwan University School of Medicine, Pyeong-dong, Jongno-gu, Seoul South Korea

**Keywords:** Spectroscopy, Electron microscopy, IOL, Opacification, Calcification, MTF, USAF target, Optical bench, IOL metrology

## Abstract

**Background:**

The opacification of hydrophilic intraocular lenses (IOLs) is a very rare complication in terms of absolute numbers. We report on the analyses of opacified Euromaxx ALI313Y and ALI313 IOLs (Argonoptics, Germany) using light and scanning electron microscopy, X-ray spectroscopy and optical bench analysis.

**Methods:**

Opacified Euromaxx ALI313Y and ALI313 IOLs were explanted after patients presented with a decrease in visual acuity. The explants were sent to our laboratory and examined using light and scanning electron microscopy. The composition of the deposits was analysed using X-ray spectroscopy. The optical quality of the intraocular lens (IOL) was assessed using the OptiSpheric IOL PRO optical bench (Trioptics GmbH Wedel, Germany). Modulation transfer function (MTF) was measured at all spatial frequencies and United States Air Force (USAF) 1951 resolution target pictures were documented.

**Results:**

Macroscopically, the entire optic was opacified in all IOLs. Light and scanning electron microscopy revealed numerous fine, granular, crystalline-like deposits, which were always distributed in a line parallel to the anterior and posterior surfaces of the IOLs. X-ray spectroscopy could prove the deposits consisted of Calcium and Phosphate. Measurements in the optical bench showed deterioration of MTF values at all spatial frequencies and the USAF target pictures demonstrated a significant reduction of brightness as well as resolution with the opacified IOLs.

**Conclusions:**

The calcification of hydrophilic IOLs only occurs rarely. The exact chemical composition of the deposits can be assessed by means of X-ray spectroscopy. Optical quality analysis of the explanted Euromaxx ALI313Y and ALI313 IOLs showed significant reduction of MTF values, which was confirmed by USAF target pictures.

## Background

The calcification of hydrophilic IOLs is a very rare complication [1]. The intracameral injection of rtPA, gas or air seems to increase the risk of opacification [2, 3]. In most cases, however, the exact cause cannot be revealed [4]. As some systemic diseases might play a role (e.g., diabetes mellitus), several processing steps during manufacturing as well as packaging material may also facilitate opacification of the optic material [4]. Impurities by silicon particles are also considered to be another potential causative factor [5].

Opacities of the optic region of the intraocular lens (IOL) can cause a significant reduction of visual acuity, contrast sensitivity or glare that can make an IOL exchange necessary. The surgical removal of an opacified IOL is often related with an increased complication rate [6]. Prior to explantation the IOL is usually cut into two or more pieces. As a consequence, the optical properties of these IOLs cannot be assessed. A material analysis is however possible in sectioned IOLs using staining methods and light and scanning electron microscopy as well as X-ray spectroscopy.

**Fig. 1:**
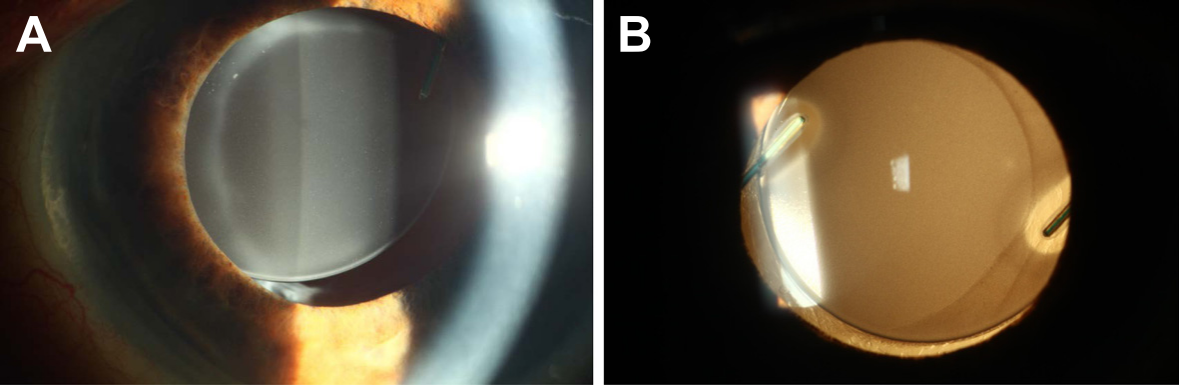
In vivo slit lamp image of a patient with an opacified ALI313 IOL. Focal **a** and retrograde **b** illumination

We report on the examination of six opacified and explanted Euromaxx IOLs from Argonoptics, Germany (five Euromaxx ALI313Y and one Euromaxx ALI313); using light microscopy, scanning electron microscopy and X-ray spectroscopy. The IOLs were all explanted due to a reduction of visual acuity which was associated with discoloration of the optic component of the lens (Fig. 1).

**Fig. 2:**
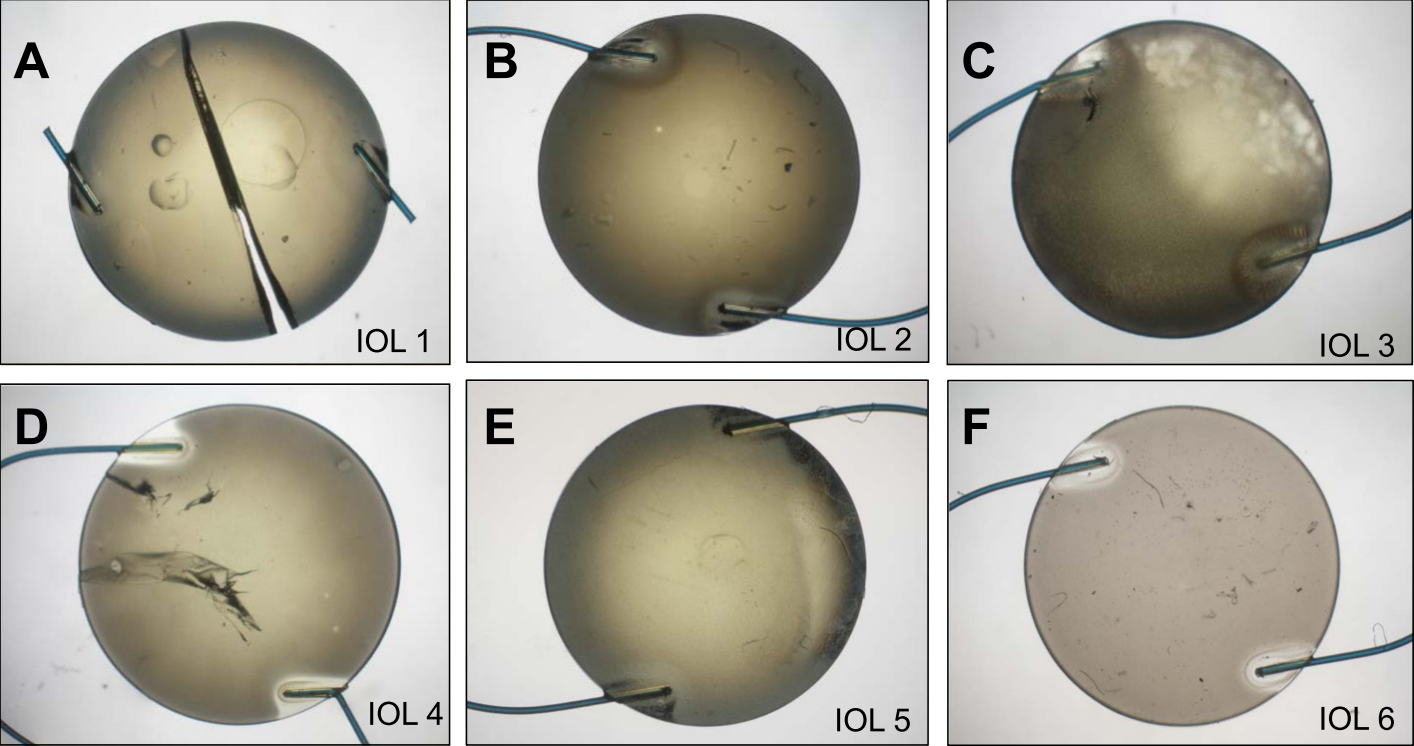
Gross examination of the opacified IOLs. **a** to **e** (IOL1 to 5): ALI313Y, **f** (IOL 6): ALI313

Optical properties of IOLs can be analysed using Modulation Transfer Function (MTF) measurements [7, 8]. IOL metrology allows qualitative and quantitative analyses of the imaging ability of a lens. We measured MTF values in IOLs which were explanted with intact optic, using IOL metrology. Furthermore, we documented United States Air Force (USAF) 1951 resolution target images of these opacified but intact lenses (Fig. 2).

## Methods

Five calcified Euromaxx ALI313Y and one ALI313 IOLs were explanted after patients complained of a decrease in visual acuity. These IOLs were sent to our laboratory and examined using light microscopy and scanning electron microscopy. Chemical composition of the deposits was analyzed by X-ray spectroscopy (EDX). The optical quality was assessed using the Trioptics OptiSpheric IOL PRO optical bench (Trioptics GmbH, Wedel, Germany).

The IOLs were examined and photographed under an Olympus BX50 light microscope with an attached Olympus C-7070 camera (Olympus Optical Co. Ltd., Tokyo, Japan).

Prior to microscopic analysis the optical quality was evaluated exemplary for one explant, which had an undamaged optic zone, using the optical bench. The Optispheric IOL-PRO optical bench (Trioptics, Wedel, Germany) was used to evaluate the optical quality parameters of IOL including Modulation Transfer Function (MTF). The MTF describing the resolution and performance of an optical system is the ratio of relative image contrast divided by relative object contrast. This technology is suggested in the ISO 11-979-2 describing the test of IOLs as the most suitable method [8]. The instrument features different types of targets as objects which are projected to the infinity through a collimator. The lens under test thus gives an image of the target at its focal plane. The measurement head composed of a microscope objective and an imaging system conjugated with a CCD camera scans through the imaging zone to find the best focus image created by the IOL tested. This image detected by the camera is then used for further analysis. The light source illuminating the target is a broad band visible spectrum light source associated with a narrow band interferential filter at 546 nm as required in the ISO norm. The image obtained via the IOL is collected by a microscope and is analyzed by the software. After image processing, an MTF curve is obtained. Using a United States Air Force (USAF) 1951 Resolution Target it is also possible to document and compare the quality of the image at a focus plane.

After optical measurement, each IOL specimen was cut in halves. One half of the IOL was stained (Alizarin Red and von Kossa stains) to determine the chemical presence nature of the deposits within the IOL. For this purpose the IOL material was treated for 2 min with 4 % buffered Formaldehyde solution and rinsed with distilled water. Subsequently the IOL-halves were placed for 3 min in an Alizarin-Red solution of 1 %. Afterwards they were rinsed again and examined by the light microscope. The specimens were then dehydrated and fixed in Paraffin, cut in 5 μm thick sagittal slices along the centre of the IOL optic and dyed according to the von Kossa method to show calcium particles. The slices of the optic material were freed of Paraffin, rehydrated with water, incubated in 5 % Silver Nitrate solution, treated with UV-light for 30 min, rinsed repeatedly with distilled water, reacted with 5 % Sodium thiosulphate and washed again before being analysed under the light microscope.

The other halves of the IOLs were used for scanning electron microscopy (SEM). For the SEM examinations the IOLs were prepared by ultramicrotomy (UCT, Leica, Germany) obtaining a very smooth cross section through the lens using a 35° diamond knife (Diatome, Switzerland). Subsequently, we examined this cross section by SEM under low voltage (<1 kV) conditions using a SU8000 microscope (Hitachi, Japan) without further coating of the sample. For a local chemical analysis, Energy Dispersive X-ray Spectroscopy (EDS) was performed using a Quantax 400 EDS detector (Bruker, Germany) attached to the SEM to detect any possible exogenous chemical elements within the IOL besides the material from which it is made.

Our laboratory study only involves analyses of IOL explants. We did not perform any additional examinations or procedures on humans. Therefore, informed consent and ethics committee approval were not required.

## Results and discussion

**Fig. 3:**
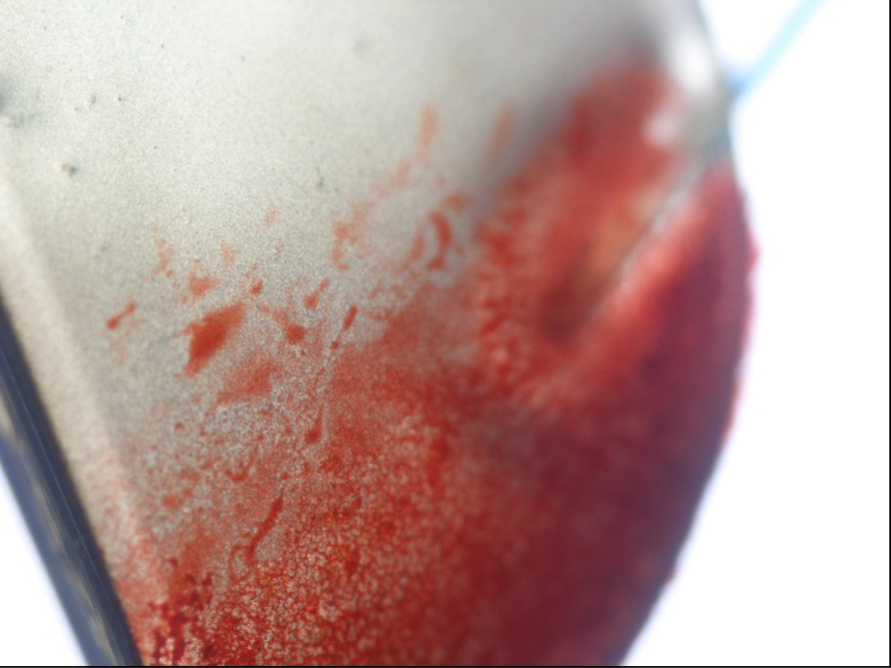
Exemplary light microscopy image of an opacified ALI313Y IOL. Staining with Alizarin Red

**Fig. 4:**
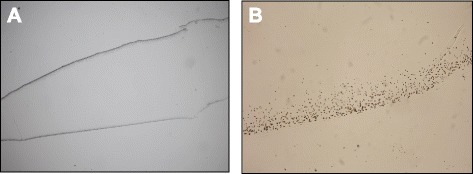
Light microscopy of the cross section of the IOLs analysed with von Kossa stain. Numerous fine, granular, crystalline-like deposits distributed in a line parallel to the anterior and posterior surface of IOLs. **a** Image showing both surfaces, **b** Image showing one surface in higher magnification

**Fig. 5:**
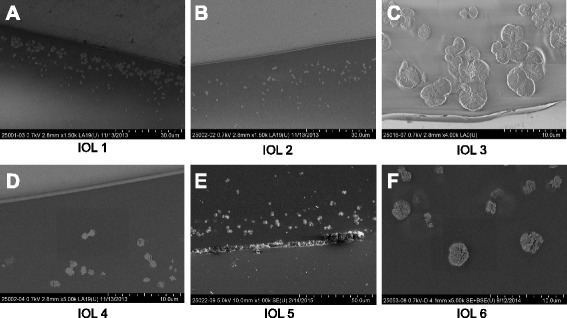
Scanning electron microscopy of the cross section of the IOLs. Numerous fine, granular, crystalline-like deposits distributed in a line parallel to the anterior (**a**, **c**, **e**) and posterior surface (**b**, **d**, **f**) of all IOLs. **a** to **e** (IOL 1 to 5) : ALI313Y, **f** (IOL 6) : ALI313

**Fig. 6:**
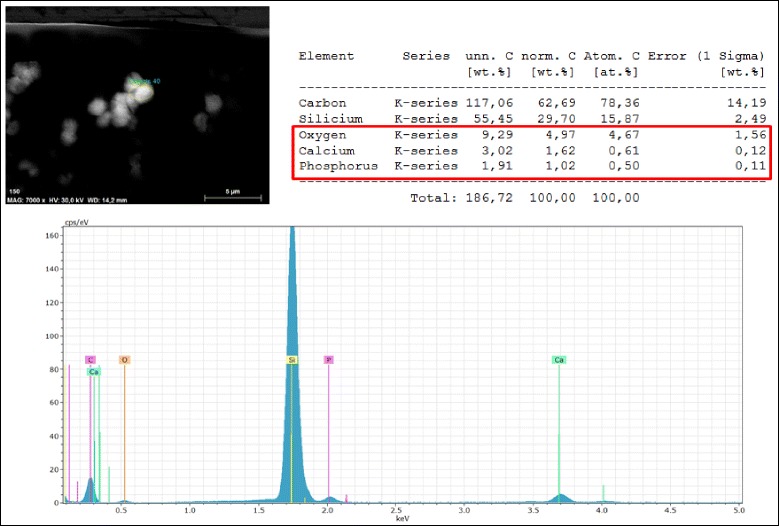
Example of X-ray spectroscopy in an opacified ALI313Y IOL. The deposits consist of Calcium phosphate. The Silicium-spike is an artefact caused by the Silicon-wafer, which is the substrate used for the analysis. Additionally, x-ray spectroscopy analysis on the surface of the IOLs (without Silicium-containing substrate) could exclude any Silicium within the precipitates

**Fig. 7:**
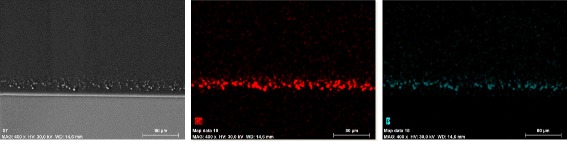
Example of element mapping in an opacified ALI313 IOL. Calcium is marked in red and phosphorus in green

Gross examination of all IOLs showed an opacification of the entire optic area (Fig. 2). The opacifications stained with Alizarin-Red were visible during light microscopy (Fig. 3). Under Light microscopy, the cross section of all IOLs (with von Kossa staining) revealed numerous fine, granular, crystalline-like deposits distributed in a line parallel to the anterior and posterior surface of all IOLs (Fig. 4). These findings were confirmed by scanning electron microscopy in all IOLs (Fig. 5). X-ray spectroscopy could prove the deposits to always consist of Calcium phosphate (Fig. 6). Element mapping supported these results (Fig. 7).

**Fig. 8:**
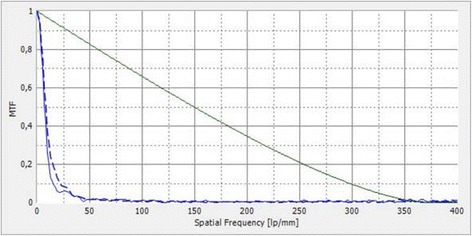
An exemplary MTF analysis of an opacified ALI313Y IOL shows significant reduction of MTF values at all spatial frequencies. The green line shows the diffraction limit. The blue lines show the measured MTF values in sagittal and tangential planes

**Fig. 9:**
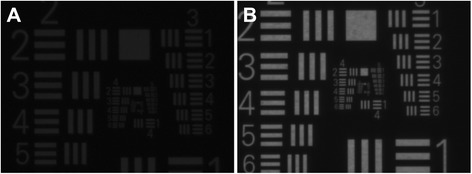
The USAF Target Analysis of the same IOL in Fig. 8. The image created by this IOL under standard light conditions shows a significant reduction of brightness and resolution **a**. Image created by the IOL with increased light **b**

The MTF measurements of the IOL with an intact optic showed a significant decrease in optical quality (Fig. 8). MTF values had deteriorated at all spatial frequencies. The USAF target analysis showed a significant reduction in brightness. After adjusting the light, however, a quite acceptable resolution could be achieved (Fig. 9). This suggests that patients may experience the impact of this type of opacification in the IOL optic as a reduction of resolution and light.

Calcification in hydrophilic IOLs has been previously reported but this is, to the best of our knowledge, the first publication about calcification of this IOL model and about the assessment of optical quality in opacified IOLs using IOL metrology [4, 5]. Different types of hydrophilic acrylic IOL materials are not equally susceptible to opacification. Therefore, material related factors seem to have a significant impact on the development of calcifications. However, the possibility of patient-related factors, such as metabolic imbalance, cannot be fully ruled out.

Izak et al., described this type of deposit in Hydroview (Bausch and Lomb, Rochester, USA) and SC60B-OUV IOLs (Medical Developmental Research Inc., Clearwater, USA) [4]. The Hydroview IOL showed granular deposits on the surface of the optic [4]. The opacification in the SC60B-OUV IOL, however, were similar to a nuclear cataract. The surface of the optic as well as the haptic region was free of Calcium phosphate deposits. Numerous granular, fine, crystalline-like deposits could be detected that were distributed in a line parallel just below the IOL surface [4, 5].

In opacified Aqua-Sense IOLs (Ophthalmic Innovations International Inc., Ontario, California) light microscopy and scanning electron microscopy analyses showed typical granular deposits on the surface the IOL [1, 4–6]. Additionally granular deposits of different sizes lined parallel to the surface of the IOL optic were found using Alizarin Red as well as the von Kossa method for staining [1, 4]. The deposits were generally located in the optic as well as the haptic region [4–6].

Aside from calcifications, other alterations of the IOL material are also described in the literature, for example IOL schisis, which is a gap formation within the IOL material [9, 10]. We did not observe this in the Euromaxx explants.

Glistenings should also be considered as a differential diagnosis in the early stage of a calcification. There are many reports in the literature about glistenings in hydrophobic acrylic IOLs, especially for the AcrySof IOL (Alcon Laboratories, Fort Worth, Texas, USA) [4, 11, 12]. However, glistenings can also be detected in other IOL materials [11]. Glistenings can be observed as early as one week after the implantation of the IOL [13]. Morphologically glistenings correlate to microvacuoles within the IOL material.

They seem to reduce the contrast sensitivity [11, 13]. However, in contrast to calcifications, a reduction of visual acuity is rare [11, 13, 14].

The explantation of the IOL is the only therapeutic option in symptomatic patients with calcified IOLs. The incidence of IOL explantation because of calcification is reported to be low. Izak et al., for example, reported in three different IOL-models with opacifcations (Hydroview, SC60B-OUV and AquaSense) the need for explantation in less than 1 % in each of the three groups [4]. The explantation of IOLs involves the risk of intra- and postoperative complications. In a series of 25 eyes with opacified Aqua-Sense IOLs, Dagres et al. reported complications (e.g., zonular dehiscence, posterior capsular rupture, corneal decompensation) in 48 % of cases [6]. Especially a strong attachment of the IOL haptics to the lens capsule makes the surgical procedure more difficult. Therefore, an indication for a surgical removal of the IOL should require thorough informed patient consent and be considered only in the case of severe complaints by the patient. It is also important not to perform a Nd:YAG-Laser capsulotomy in eyes with an opacified IOL as that can increase the complication rate during an IOL exchange procedure.

In this study, unfortunately we could not evaluate all IOLs using optical quality assessment, because some IOLs were explanted after bisection during surgery. Additional studies are needed to understand the relationship between the clinical characteristics of opacification and the results of analyses on optical bench systems as well as to determine possible patient-related factors (e.g., metabolic disorders) as causes for the opacification of this IOL model.

## Conclusions

The calcification of hydrophilic IOLs is a rare complication. Granular deposits under the surface of the Euromaxx ALI313Y and ALI313 IOL lenses can cause a degradation of lens optical quality and reduction of visual acuity.

The calcium deposits can be demonstrated using the von Kossa staining-method or staining with Alizarin-Red. The exact chemical composition of the deposits can be assessed using X-ray spectroscopy.

The Argonoptics company has reported that it uses hydrophilic acrylic blanks from a different polymer manufacturer since January 2012. According to Argonoptics, there has been no case of opacification with the new material so far (Personal communication).

### Availability of supporting data

The data on which the conclusion of the paper relies are included in the manuscript and presented in form of figures.

## Abbreviations

IOL: intraocular lens
IOLs: intraocular lenses
MTF: Modulation transfer function
USAF target: United States Air Force target
SEM: scanning electron microscopy
